# Exploring the multifaceted factors influencing overweight and obesity: a scoping review

**DOI:** 10.3389/fpubh.2025.1540756

**Published:** 2025-04-09

**Authors:** Mohsen Jalilzadeh, Salime Goharinezhad

**Affiliations:** Department of Health Services Management, School of Health Management and Information Sciences, Iran University of Medical Sciences, Tehran, Iran

**Keywords:** obesity, overweight, socio-cultural determinants, economic factors, technological influences, environmental factors, public health policy, scoping review

## Abstract

**Introduction:**

Obesity and overweight problems in public health have substantial impacts which affect the health status of individuals and community well-being and healthcare service provision worldwide. This scoping review aims to identify and classify factors from social, technological, environmental, economic and political domains which influence obesity and overweight conditions. The systematic analysis of determinants in this study generates usable information to guide public health intervention design and obesity epidemic management strategies.

**Methods:**

The study utilized the ProQuest, ISI Web of Science, PubMed, and Scopus databases, and it also included grey literature in its analysis. The research objectives focused on identifying factors that contribute to overweight or obesity issues. The researchers used framework analysis to examine the qualitative data collected from these studies.

**Results:**

The synthesis incorporated 121 research studies which satisfied the established criteria. This comprised 98 studies from 46 different countries, 17 studies conducted at the international level, and 6 studies involving multiple countries. Eighty-two factors influencing overweight and obesity were identified as determinants and categorized into five main categories: sociocultural, economic, technological, environmental, and political. Most of the identified determinants belong to the socio-cultural category, which demonstrates their substantial impact on lifestyle and health behaviors.

**Conclusion:**

The implementation of public health prevention and intervention programs depends on complete knowledge of all factors that affect overweight and obesity rates. This issue needs a comprehensive approach which analyzes sociocultural aspects together with economic, technological, environmental, and political factors, as well as other policy goals within defined societal challenges. Effective solutions to resolve this situation depend on multi-sectoral collaboration to tackle obesity and promote health-enhancing factors for the entire community.

## 1 Introduction

Overweight and obesity represent preventable health issues with multiple factors that have become prevalent in more than one-third of the global population worldwide (1–4). These conditions characterized by abnormal or excessive fat accumulation that poses a risk to health (5). A body mass index (BMI) over 25 is classified as overweight, while a BMI over 30 is classified as obese (2). The health risks from overweight and obesity exist, yet obesity leads to more severe and more frequent chronic diseases than overweight (5).

The issue represents a worldwide health concern which affects life quality throughout various population groups (6–9). Worldwide, at least 2.8 million people die annually as a result of overweight or obesity (10). The link between obesity and high-income countries used to exist (9–11) but the condition has become widespread throughout low- and middle-income countries as well (10–12). Overweight and obesity rates keep increasing at an alarming rate. Research shows that childhood and adolescent obesity rates among 5 to 19 years old increased four times between 1990 and 2022 from 2 to 8%. Similarly, the prevalence of obesity among adults aged 18 and older more than doubled, increasing from 7 to 16% (10). A total of 2.6 billion people suffered from obesity and overweight conditions during 2020. The global prevalence of overweight and obesity rates will rise from 38% in 2020 to surpass 50% in 2035. It is predicted that more than 4 billion individuals will experience overweight or obesity by 2035 (13). Specifically, the prevalence of obesity is expected to rise from 14 to 24 percent of the population over the same period, and nearly 2 billion adults, children and adolescents will be impacted by 2035 (13).

Overweight and obesity serve as main factors that lead to noncommunicable diseases, including cardiovascular disorders, diabetes, and cancer. These conditions lead to more than 5 million annual deaths with most fatalities happening before age 70 (3). Obesity-related health risks increase mortality rates and lead to severe reductions in quality of life through physical disabilities, as well as psychological conditions such as depression, self-esteem issues, and social discrimination. Because of their long-term nature these conditions trigger ongoing healthcare issues which create an enduring healthcare burden throughout the world (14, 15).

Healthcare costs associated with obesity treatments and their complications along with diminished productivity and earlier retirement and elevated mortality rates constitute the economic impact of overweight and obesity (13, 15–17). The projected costs for healthcare services will elevate from $1.96 trillion in 2020 to exceed $4 trillion by 2035. Furthermore, the economic effects of obesity will reduce global Gross Domestic Product (GDP) between 2.4 and 2.9% during the period from 2020 to 2035 (13). The economic decline during this period matched the estimated effects of the COVID-19 pandemic on the world economy during its most difficult year in 2020 that reduced GDP by 3% (13). The World Health Organization (WHO) has declared obesity as a significant public health emergency which demands swift worldwide intervention (2, 7, 15). Tackling the rise in obesity is vital for achieving the Sustainable Development Goals (SDGs) (3, 18–20). The SDG Target 3.4 sets a goal to decrease premature deaths from non-communicable diseases until 2030. The solution to the obesity crisis lies in addressing the root causes of obesity factors that are rapidly creating a global environment conducive to obesity (1).

Literature about obesity and overweight determinants establishes both genetic and biological elements as substantial components of this research field. However, Literature lacks comprehensive exploration of social, economic, environmental, technological and political aspects regarding these conditions. Therefore, to address this gap in knowledge, it seems necessary to conduct a scoping review. In order to combat the increasing prevalence of obesity and overweight, this scoping review systematically explores the social, technological, environmental, economic and political factors that increase obesity and overweight occurrences. The review establishes a complete framework through an analysis of current research about these determinants to support public health intervention planning and policy development. The holistic approach provides essential knowledge needed to develop effective strategies which address the worldwide obesity epidemic.

## 2 Methodology

The research methodology built by Arksey and O’Malley (21), received further development by Levac et al. (22), and followed Joanna Briggs Institute (JBI) protocols (23). A scoping review includes different study designs and contexts to create an organizational structure of existing research within a particular field and follows a five-step execution process (21):

1) Identifying the research question
2) Searching for relevant studies
3) Selecting studies
4) Charting the data
5) Collating, summarizing, and reporting the results

This paper discusses these stages in accordance with the current scoping review framework.

### 2.1 Step 1: Identifying the research question

The research question for this review study was defined as follows:

- Which factors from socio-cultural, economic, technological, environmental and political categories influence overweight and obesity?
- How does each category of factors contribute to the rising prevalence of overweight and obesity?
- What are the global trends in obesity research, and how do geographical and socioeconomic differences affect the understanding of obesity factors?
- What methodological approaches have been used in obesity research, and how can future studies improve the assessment of overweight and obesity factors?
- What gaps exist in our current understanding of the factors of overweight and obesity, and which future research should receive priority for optimizing obesity prevention and intervention approaches?

### 2.2 Step 2: Searching for relevant studies

The literature review included research from 2013 to December 2023 which was retrieved from electronic databases such as PubMed, Scopus, ISI Web of Science, and ProQuest. The search strategy utilized Boolean operators and key terms such as *factor*, *determinant*, *driver*, *obesity*, *overweight*, *social*, *technological*, *environmental*, *economic*, and *political*. The search strings were specifically designed for each database to maximize the retrieval of relevant studies, using the example shown in Table 1 for the PubMed search strategy. The initial search was performed on January 31, 2023, and an updated search was completed on April 7, 2023. To enhance comprehensiveness, backward and forward snowballing was employed by screening the reference lists of included studies.

**Table 1:** Search strategy for the PubMed database.

Search string record
((“Social”[Title/Abstract] OR “cultural”[Title/Abstract] OR 1231 “socio-cultural”[Title/Abstract] OR “Technological”[Title/Abstract] OR “Economic”[Title/Abstract] OR “Political”[Title/Abstract] OR “ecological”[Title/Abstract] OR “environmental”[Title/Abstract]) AND (“factor”[Title/Abstract] OR “determinant”[Title/Abstract] OR “driver”[Title/Abstract]) AND (“obesity”[Title/Abstract] OR “overweight”[Title/Abstract]))

### 2.3 Step 3: Selecting studies

**Figure 1:**
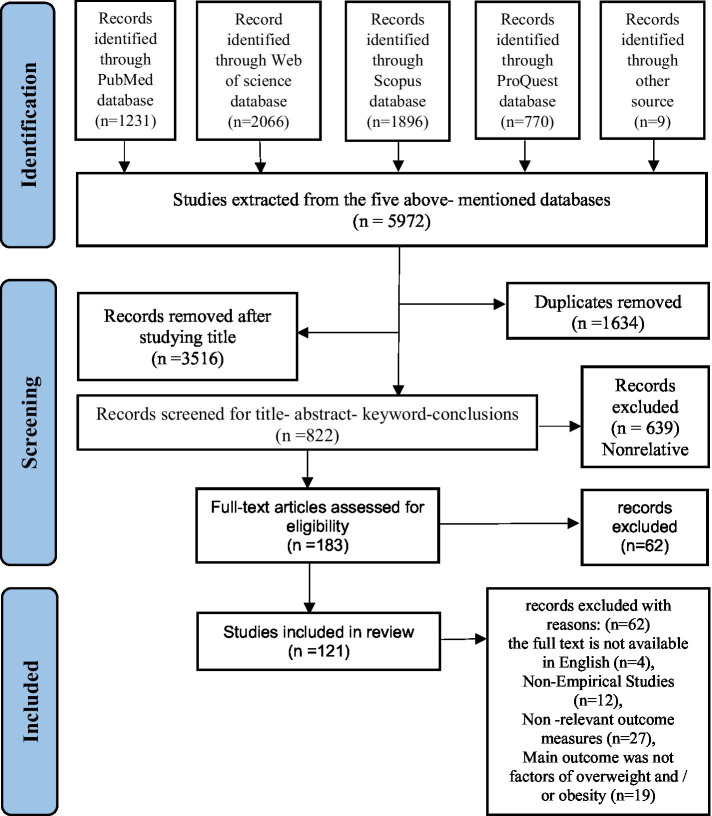
PRISMA flow diagram of the included studies.

The selection process is detailed in the Preferred Reporting Items for Systematic Reviews and Meta-Analyses (PRISMA) flowchart (Figure 1) (24), which illustrates the number of records identified, screened, excluded, and included in the review. The study selection process was managed using EndNote 21 to eliminate duplicates. The screening process consisted of two distinct stages for selection:

- A single researcher (M.J.) performed title and abstract screening as part of the relevance evaluation process to determine inclusion consistency with established criteria.
- Two reviewers (M.J. and S.G.) conducted separate evaluations of all potentially eligible studies in their full-text format. The reviewers reached agreement about any conflicting interpretations through discussion until they achieved consensus.

### 2.4 Eligibility criteria

The Population, Concept, Context (PCC) framework by JBI served to establish the eligibility criteria for this scoping review (25). The JBI recommendation of the PCC framework enables researchers to develop clear research questions while identifying suitable studies through its three essential elements:

- **Population (P)**: The research targets specific individual groups known as the population.
- **Concept (C)**: Relates to the primary phenomena of interest.
- **Context (C)**: The research environment where studies take place represents context.

Table 2 presents the complete eligibility criteria which the review team developed by conducting an initial exploratory search and subsequent discussions.

**Table 2:** Eligibility criteria.

Inclusion criteria	
Source of information	Peer-reviewed journals and publicly available grey literature
Language	Studies published in English
Time frame	2013 to December 2023
Context	Global, without geographical restrictions
Study population	All age groups and genders
Phenomena of interest	Socio-cultural, technological, economic, environmental and political factors or determinants that influencing obesity or overweight
Type of studies	Any study that examined determinants or factors associated with obesity or overweight
Study Design	Quantitative, qualitative, mixed-method studies, and systematic reviews
Primary Outcomes Measured	Overweight and obesity classified according to the BMI index based on the World Health Organization (WHO) scale (2)

Exclusion criteria	
Phenomena of interest	Genetics and biological factors related to overweight and obesity
Type of studies	Methodological studies, discussion papers, general literature reviews, Case reports, case series, letters to editors, commentary pieces and study protocols
Excluded Outcome Measures	overweight and obesity based on other index (Waist circumference, Waist-to-hip ratio, Skinfold thickness) and not in accordance with the WHO scale
Language	Non-English studies
Time restriction	Before 2013

### 2.5 Step 4: Charting the data

A standardized data extraction form built in Microsoft Excel was used for the data collection process. The researchers extracted the following information from each study:

- Study title
- Author(s) and publication date
- Country of origin
- Population characteristics (age, gender)
- Study design
- Study aims and research questions
- Key findings, factors, and determinants associated with obesity

M.J. and S.G. independently checked 20% of the studies to verify data extraction accuracy and consistency. The reviewers addressed any discrepancies by discussing them until both parties reached agreement. For additional details, the complete charting form is available in the Appendix section.

### 2.6 Step 5: Collating, summarizing and reporting the results

The research team analyzed extracted data using thematic analysis and an analytical framework to identify consistent patterns and relationships between the five Social, Technological, Economical, Environmental and Political (STEEP) categories. The research incorporated both qualitative and quantitative data findings to present a whole picture of obesity determinants. The thematic synthesis enabled researchers to uncover important factors while studying their relationships to one another.

### 2.7 Validity and reliability

This review adopted steps to guarantee its reliability and rigor, despite the typical absence of formal quality assessment for included studies in scoping reviews:

- The search strategy developed in partnership with an information science expert to achieve both high levels of inclusiveness and reduce potential bias.
- Data extraction performed independently by the reviewers, and any disagreements were resolved through discussion and agreement.
- The review team documented all methodology steps transparently to enhance the ability to replicate the findings.

## 3 Results

**Figure 2:**
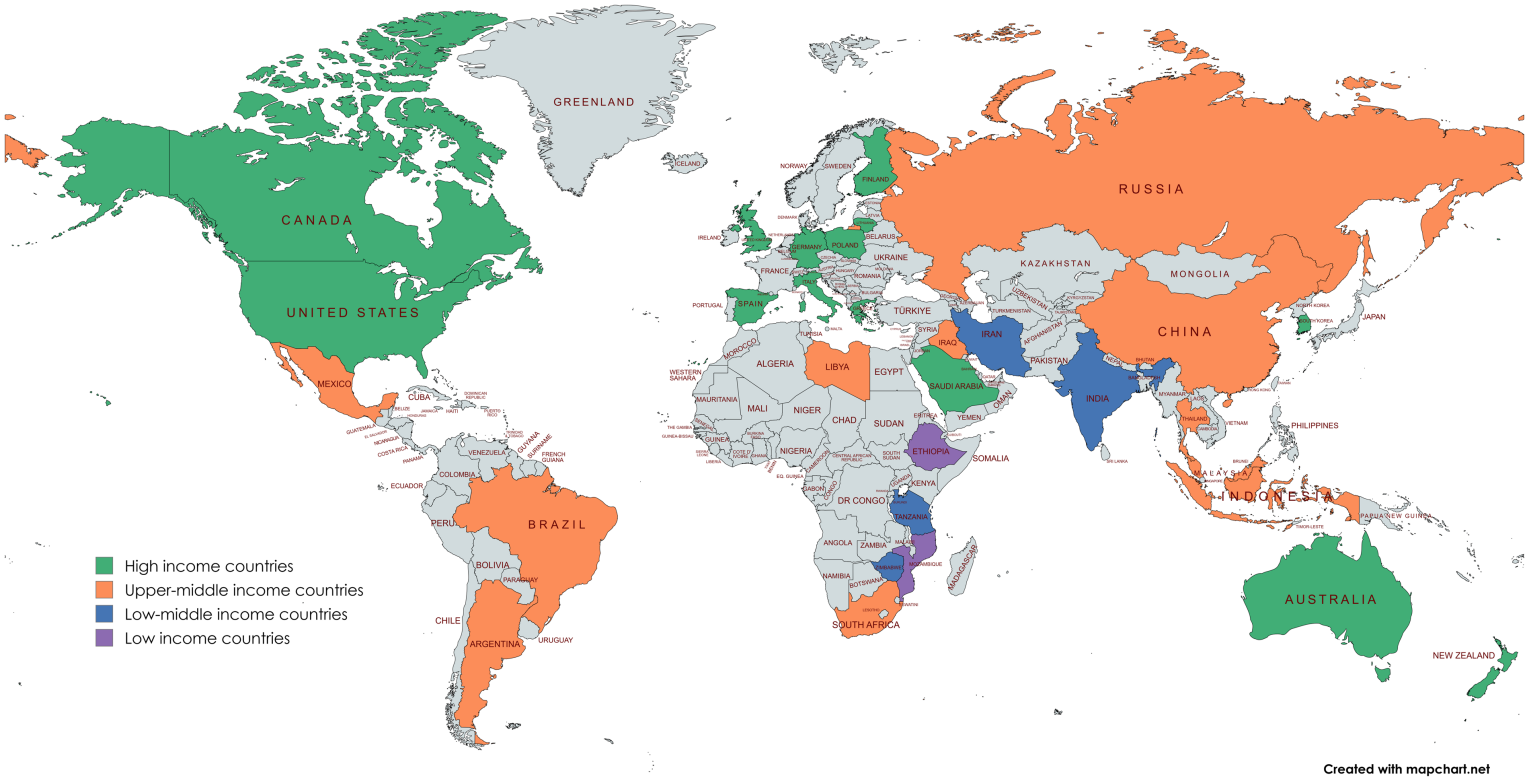
Geographical distribution of studies.

**Figure 3:**
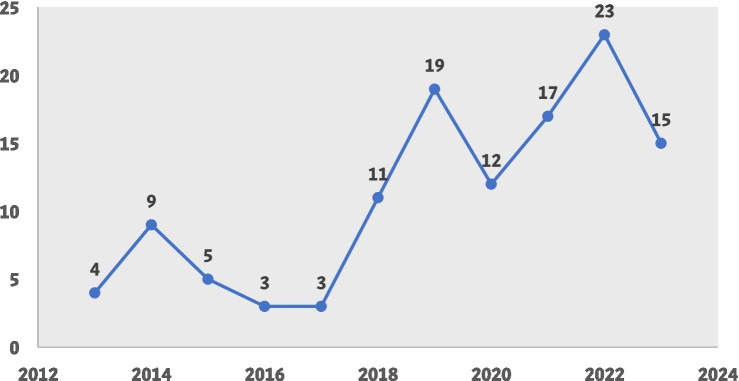
The time trend of included studies.

The characteristics of the studies included in the qualitative synthesis are summarized in Table 3, which details the geographic distribution, study design, and demographic focus of the included studies. As depicted in Figure 2, most research was conducted in North America, with the United States leading in the number of studies. A total of 30 studies (24.8%) which made up the majority were performed in North American territories. Studies from the United States made up the largest group, totaling 24 (19.8%; see Figure 2). Excluding globally conducted research (23 studies, 19%), the rest of the studies fell into three categories: 44.6% in high-income countries, 19% in upper middle-income countries and 12.4% in low middle-income countries. The research data show that studies conducted in low-income countries account for only 5% of the total investigations, as shown in Figure 2. The majority of research (61.2%) within the entire dataset employed a cross-sectional study design. Figure 3 shows that obesity-related research has experienced significant growth from 2019 to 2023. Most research studies were performed in 2022 according to the analysis of publication dates. Additionally, 36 studies (29.8%) were published between 2013 and 2018, while 85 studies (70.2%) were published from 2019 to 2023 (see Figure 3).

**Table 3:** Characteristics of the studies included in the qualitative synthesis.

Characteristic	No. of studies (percent)
Continent	
North America	30 (24.8%)
Asia	28 (23.1%)
Europe	20 (16.5%)
Africa	17 (14.1%)
International	17 (14.1%)
Australia	5 (4.1%)
South America	3 (2.5%)
Oceania	1 (0.8%)
Publication year	
2013–2014	13 (10.7%)
2015–2016	8 (6.6%)
2017–2018	15 (12.4%)
2019–2020	29 (24%)
2021–2022	41 (33.9%)
2023	15 (12.4%)
Sex Distribution	
Both	106 (87.6%)
Women	12 (9.9%)
Nonreported	3 (2.5%)
Method	
Cross-sectional	74 (61.2%)
Review	23 (19%)
longitudinal studies	12 (9.9%)
qualitative studies	11 (9.1%)
Mixed-Method	1 (0.8%)
Sample size	
<100	8 (6.6%)
101–1,000	22 (18.2%)
1,001–10,000	29 (24%)
10,001–100,000	23(19%)
> 00,000	22 (18.2%)
Nonreported	17 (14%)
Age group distribution	
Adults	49 (40.5%)
Children	22 (18.2%)
Children and adolescents	18(14.8%)
All age	12 (9.9%)
Adolescents	8(6.6%)
Adolescents and adults	6(5%)
Nonreported	6 (5%)

Adults received the most attention in obesity research studies, as 40.5% (49 studies) focused on this age group. Most research (106 studies, 87.6%) examines gender-specific patterns and determining factors that affect both men and women but demonstrates additional research interest in female groups.

**Figure 4:**
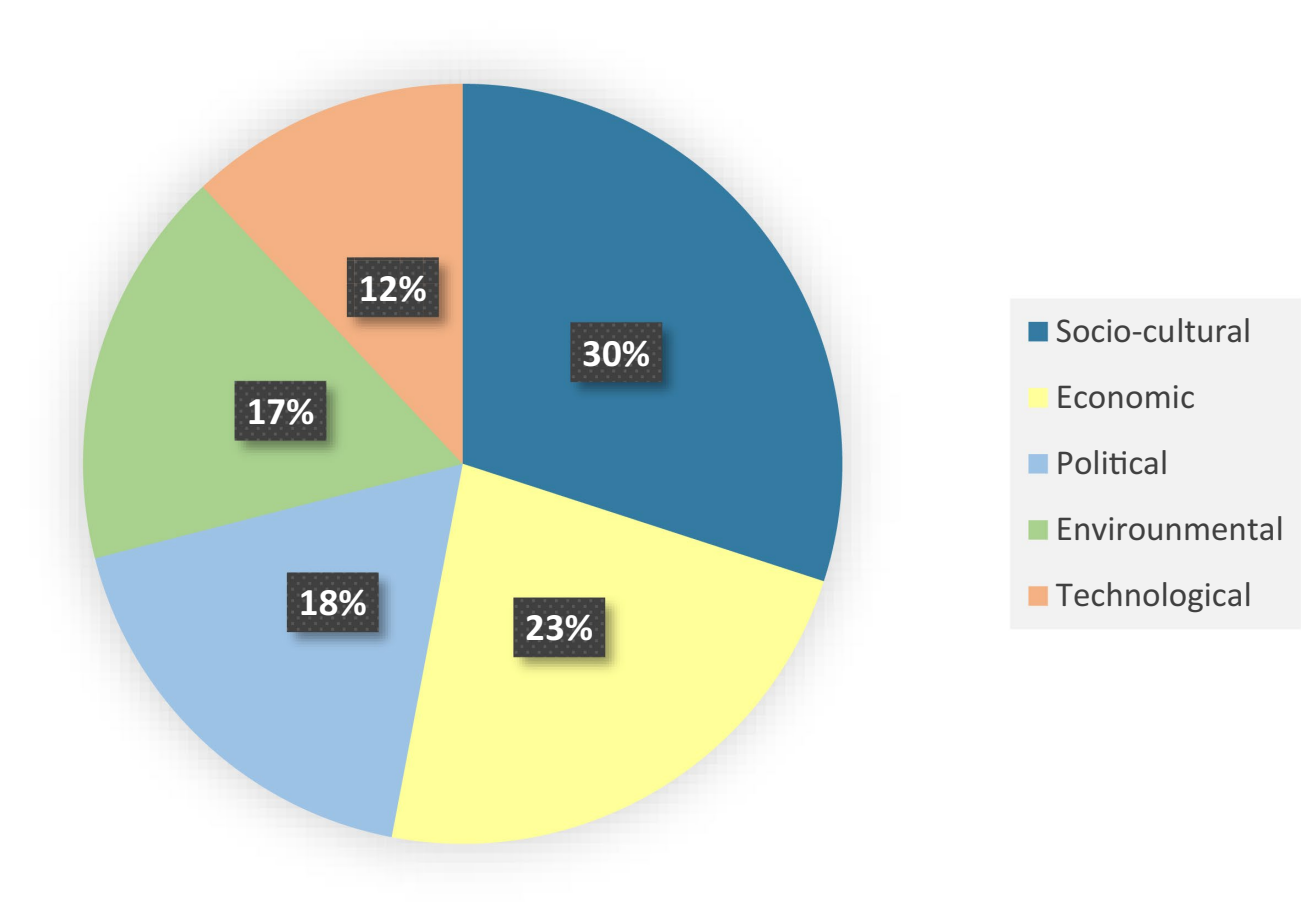
The percentage of determining factors based on the STEEP framework.

The factors influencing obesity, categorized according to the STEEP framework, are presented in Table 4. The analysis identified 82 different factors that fall under the five main categories of the STEEP framework. The majority of factors fell under the socio-cultural category (*n* = 24), whereas the technological category contained the fewest factors (*n* = 10) (see Figure 4).

**Table 4:** Factors influencing obesity categorized by STEEP framework.

Category	Factors
Socio–cultural	**Socio-demographic Factors:** (Age, Gender, Marital Status, Education Level) **Socioeconomic Status (SES):** (Income Levels, Employment Status, Urban or Rural Residence) **Social inequality** (disparities in income, education, and opportunities) **Family structure and size** (single-parent households, number of siblings) **Parental education and employment** (impact on children’s dietary habits) **Peer and social support networks** (influence of friends, community support) **Early childhood development** (socioemotional learning, childhood adversity, peer interactions, Infant-feeding method) **Body weight stigma and discrimination** (negative societal attitudes toward overweight individuals) **Screen time and sedentary activities** (time spent on TV, computers, and video games) **Social norms around food** (e.g., food as reward, bonding or way to express love, family meals) **Parental perceptions and influence on child body weight (**parental attitudes toward child’s weight and health) **Peer and family influence** (e.g., family diet habit, family income, family-related stress) **Social support**) e.g., community connection, global happiness, civic engagement, collective efficacy) **Social globalization** (exposure to global cultural norms through media and migration) **Social Opportunities** (Access to education, health insurance, access to health services, health literacy, Perceived discrimination) **Crime rate** (impact of unsafe neighborhoods on outdoor physical activity) **Human Development Index** (HDI) (a summary composite measure of a country’s average achievements in three basic aspects of human development: health, knowledge and standard of living) **Women’s empowerment** (gender equality and influence on health choices) **Ethnic, racial and religious differences** (cultural attitudes) **Gender roles affecting dietary choices and social expectations** (expectations influencing eating habits) **Cultural perceptions of obesity** (as a symbol of wealth or prosperity) **Beliefs about health and body weight** (cultural attitudes towards ideal body types) **Dimensions of national culture** (e.g., individualism, uncertainties or ambiguities, focus on freedom and happiness, focus on short-term goals, intolerant of inequalities and power hierarchies, less competitive and more feminine) **Dietary rituals and habits** (traditional food practices and meal patterns)
Technological	**Influence of digital and social media on body image and diet choices** (e.g., exposure to ideal body types influencing self-perception) **Increased screen time and digital entertainment** (e.g., watching TV, using computers, playing video games) **Online food delivery services promoting processed foods** (e.g., fast food delivery apps increasing access to unhealthy options) **Dependency on passive transportation and reduced physical activity** (e.g., reliance on cars reducing physical activity) **Work-from-home and remote learning effects on sedentary behavior** (reduced movement due to telecommuting) **Increased access to technology for entertainment and digital advertising** (e.g., internet, social media) **Influence of digital advertising on dietary choices** (e.g., targeted ads promoting unhealthy foods) **Decrease in physical activity due to automation and modern conveniences** (e.g., household gadgets reducing manual work) **Digital advertising and targeted marketing** (impact of online ads on consumer behavior) **Widespread expansion of online educational programs** (reduced physical activity due to increased online learning)
Economic	**National income per capita** (association with obesity prevalence in high- vs. low-income countries) **GDP** (gross domestic product as a marker of national wealth and health outcomes) **Decreasing purchasing power for healthy foods** (rising costs of nutritious foods) **Price index of food items** (impact of food pricing on dietary choices) **Poverty** (limited access to nutritious foods) **Prolonged Financial stress** (impact on eating habits and stress-related weight gain) **Economic shocks** (e.g., inflation, recession affecting food security) **Unemployment rate** (association with reduced access to healthy foods) **Job insecurity** (stress-related eating and reduced income for healthy food) **Gini coefficient** (measure of income inequality and its correlation with obesity) **Urbanization and rural residence disparities** (differences in food availability and physical activity opportunities) **Calorie intake per capita** (link between economic growth and increased caloric consumption) **Cost of exercise** (affordability of gym memberships and fitness activities) **Domestic expenditure on health and food** (impact of food pricing on dietary choices) **Housing insecurity** (impact on diet quality and food access) **Food insecurity and limited access to nutritious options** (association with high-calorie, low-nutrient diets) **Economic liberalization and trade effects on diet** (association with availability and quality of diet) **Costs of nutritious vs. processed foods**)The high cost of nutritious and healthy foods( **Access to healthy foods based on economic constraints** (limited access to nutritious foods)
Environmental	**Climate and Geography** (temperature, harsh winter climate, humidity, latitude, dust) **Global warming and climate change limiting outdoor activities** (impact on outdoor physical activity and food supply) **Traffic** (e.g., air quality and traffic-related barriers to physical activity) **Pollution** (air, light, obesogenic chemicals with endocrine-disrupting properties in food production, packaging and environment, heavy metals, maternal second-hand smoking) **Built environment** (e.g., Infrastructure for active living, quality of roads, street connectivity, public transport, residential density) **neighborhood food environment** (availability of healthy food options in local stores) **Access to parks, green spaces, and recreational facilities** (impact on physical activity levels) **Neighborhood walkability and safety** (e.g., crime rates affecting willingness to exercise outdoors) **Availability of sidewalks, bike lanes, and traffic infrastructure** **Climate change impacting food supply** (e.g., availability of fresh produce) **COVID-19 pandemic effects on diet and physical activity** (lockdowns increasing sedentary behavior) **Overpopulation and reduced access to recreational areas** **Urban vs. rural residence** (differences in access to food and recreation) **Neighbourhood deprivation** (impact of socio-economic status on local food and exercise environments)
Political	**National Obesity Strategy policies** (government frameworks targeting obesity prevention) **Influencing policymaking processes through lobbying** (e.g., through lobbying, “Big Food” has directly sought to influence policy and governance). **Government policies on food and physical activity** (Healthy food production and distribution policies, encouraging the reformulation of processed foods to be healthier, setting policies for food vendors, health regulations on the numbers, availability and distribution of high-calorie fast food restaurants ( **Trade liberalization** (impact of global food trade on local diets) **School nutrition and physical activity policy** (e.g., mandating healthy school lunches, physical education programs) **Policy response to reduce the impact of advertising on children** (e.g., restrictions on marketing unhealthy foods to minors) **Regulations on food labeling and health standards** (mandatory calorie labeling on menus) **Policies supporting active transportation and recreation** (investment in public transport and green spaces) **Urban planning regulations** (e.g., green spaces, bike lanes) **Public health campaigns for obesity prevention** (government-sponsored initiatives promoting healthy lifestyles) **Taxation of unhealthy foods** (e.g., sugary beverage taxes to reduce consumption) **Subsidizing healthier and less dense foods** (financial incentives for purchasing nutritious food) **Urbanization and development policies** (shifts in dietary patterns and physical activity levels) **Food marketing and advertising regulations** (e.g., reduce advertisements for unhealthy foods) **Insufficient policies promoting physical activity in schools and communities** (Facilitating free exercise)

Out of 121 studies, 90 (74.4%) explored the relationship between socio-cultural factors and obesity or overweight. Among these, 59 studies focused on socio-demographic factors and their influence on overweight or obesity, with a consensus that these factors are of significant importance. Notably, age, gender, education level, residential areas, and marital status were identified as key factors associated with overweight and obesity, as reported in more reviewed studies. Another significant influencing factor for overweight and obesity, categorized under socio-cultural aspects, is the impact of family. The relationship between family influence factors and overweight and obesity was analyzed in 36 studies. The most notable factors associated with family influence, whose effects on the overweight and obesity of children were reported in multiple articles, include family income, parental education, and occupation. Twenty-six research studies examined the relationship between economic factors and overweight or obesity. Among these studies, the unemployment rate, low income, and GDP were identified as significant factors linked to overweight and obesity, with the highest occurrences reported in the article. Twenty-two studies analyzed technological factors in the development of overweight or obesity, confirming technological progress as a primary cause.

The most significant factors associated with technological advancement included television, the internet, video games, and social media, as well as digital marketing and advertising. Thirty-nine studies were reviewed to analyze the relationship between environmental factors and overweight or obesity. The most significant factors identified within the environmental category were the neighborhood food environment and the built environment, as reported in the majority of the articles. Eighteen research studies examined the association of political factors with overweight or obesity. The most prominent factors linked to overweight and obesity were food and nutrition policies, along with school food and physical activity policies, were the most prominent factors linked to overweight and obesity, as reported in most articles.

## 4 Discussion

This scoping review shows that overweight and obesity result from many intertwined factors which create such a complex issue. The research analysis detected 82 contributing factors that researchers grouped into five fundamental categories: socio-cultural, economic, technological, environmental and political. The most important factors leading to overweight and obesity stem from the socio-cultural category which demonstrates how family structures and social connections together with cultural values play a crucial role in these conditions. Research shows that economic factors, along with advances in technology and environmental changes especially urbanization have a great impact on obesity patterns. Public health policies together with regulatory frameworks both serve as essential political factors that influence this problem.

Current global studies about obesity and overweight demonstrate that these problems exist throughout worldwide regions. The diverse geographical mapping of overweight and obesity research provides analysts and policymakers with enhanced knowledge about global challenges and solution possibilities although low-income and lower-middle-income countries have fewer studies.

Analysis of the distribution of research approaches used in obesity and overweight studies showed that various methods were used throughout the series, but cross-sectional studies were the predominant method, with 74 studies (61.2%) using a cross-sectional method in their studies. Research on obesity needs to establish longitudinal studies because its complex causes require long-term observation of weight change and other contributing factors. This research design allows researchers to detect connections between multiple influencing factors. Additionally, research that relies on qualitative methods which use lived experiences from individuals produces substantial understanding of the subject. Furthermore, a combination of quantitative and qualitative research approaches through mixed methods analysis provides an extensive examination of obesity and overweight causes and effects and intervention solutions. Based on age group distribution, the majority of studies concentrated on adults (40.5%). It appears essential to conduct further research, especially concerning the children and adolescents age group. However, upon reviewing the history of these studies, it was discovered that 15 out of 22 studies (68%) in the children’s age group were carried out in the last 5 years, from 2019 to 2023. This indicates the growing significance of research on obesity and overweight in children in recent years.

The socio-cultural category was the most dominant, as it encompassed a wide range of identified factors. The research demonstrates how sociodemographic factors together with family dynamics and social support systems, and cultural norms, directly affect weight-related behaviors and their corresponding results. The research analyzed age, gender, marital status, education level and socioeconomic status as socio-demographic factors. Multiple studies across the literature identified these factors as important variables. Education together with gender along with age and marital status and socioeconomic factors create a complex relationship that proves the need for specific interventions to fight obesity.

Research findings demonstrate that age has a positive relationship with obesity and overweight because BMI tends to rise as people grow older (26–57) while older age groups (36, 43, 49, 52) may show a decline. Research revealed that people under 35 years old demonstrated both a higher BMI transformation rate and a greater number of obesity and overweight cases (58). Maternal age during pregnancy has been recognized as a significant influencing factor in the likelihood of obesity in children, with offspring of mothers over 30 years old at the time of pregnancy being at a higher risk of developing obesity (26). Consequently, factors such as population aging, early obesity interventions due to the rapid increase in obesity rates among younger age groups, and later marriages and pregnancies among women should be considered as crucial age-related elements in the design and implementation of obesity interventions.

The literature demonstrates that women experience more BMI changes and show higher obesity rates than their male counterparts in multiple research studies (27–44), yet alternative findings exist (26, 45–49). Male individuals tend to experience higher rates of being overweight, while female individuals exhibit increased rates of obesity (50). The combination of sedentary work activities with dietary choices plays a significant role in creating weight differences between women and men in developed nations. The physical activity patterns in developing countries have undergone significant changes, which specifically affect women. The sedentary lifestyle of women compared to men makes them more susceptible to weight gain from energy-dense foods. Sociocultural beliefs and values related to physical activity and body weight have a more significant impact on gender differences in overweight and obesity in developing countries compared to developed countries (51).

The majority of studies reviewed indicate that being married is associated with a higher risk of overweight and obesity compared to being single or unmarried (30–32, 34, 36, 38, 50, 52–58). However, some research have reported contrasting outcomes to this general trend (59, 60). Additionally, people who are divorced or widowed experience more chances of being overweight or obese than individuals who remain single (38). Single individuals usually take weight management seriously so they can appear more appealing to romantic prospects, yet married people tend to spend less time being active because they have more household responsibilities. After marriage, people tend to experience changes in their eating habits that create obstacles for weight management (61).

Education level, alongside economic status determines how people choose their food and how active they remain. The way educational background affects obesity shows diverse patterns depending on gender and age, along with socioeconomic situations. Studies have produced contradictory data about the connection between educational attainment and obesity prevalence. Research shows that higher education acts as a protective measure against overweight and obesity, while this pattern mostly occurs in younger and male populations (36–38, 49, 50, 57, 60, 62–67).

Conversely, other research has discovered that people with higher education levels tend to overweight and obesity problems more frequently and this impact is particularly strong for women (31, 33, 52, 53, 68–71). The relationship between education level and weight status can be affected by other variables such as income and occupation (27, 54, 67, 72). Research discrepancies about education and weight status emerge from different socio-cultural factors alongside economic and environmental conditions which affect this connection. People with advanced education benefits from better knowledge about healthy choices, yet face dual risks from inactive work settings and excessive high-calorie processed foods found in their neighborhoods.

The review demonstrates how living in urban or rural areas affects the prevalence rates of overweight and obesity. The studies consistently have indicated that residing in urban or metropolitan areas was associated with a higher risk of being overweight or obese compared to living in rural areas (31, 33, 34, 38, 47, 50, 52, 54, 68, 73–76). Some studies have also suggested that the urban–rural gap is narrowing due to the increasing prevalence of overweight and obesity among rural residents (28, 29, 70, 77–79). Rural food environments alongside lifestyle patterns have undergone a shift because unhealthy food choices have become more accessible and people spend less time engaging in physical activities.

Most factors related to the family influence factor were those that contributed to obesity and overweight in children and adolescents. Research shows that children whose parents have advanced education tend to maintain healthy body weight (73, 80–86). Research indicates that parents with higher education unintentionally guide their children toward a high-energy sedentary lifestyle, which raises their weight problem risk (66, 87). These findings demonstrate the intricate relationship between parental education and child weight status because protective and risk factors were discovered. It seems that additional investigation will be needed to explain the fundamental processes and place-specific factors which regulate this connection. Parental occupational status, including maternal employment (54, 66, 84, 85), dual parental employment (82, 85–87), and high parental occupational level (73, 82), has been reported to be increasingly linked to childhood obesity.

Another significant factor that numerous studies have indicated influences childhood overweight and obesity is family eating habits. These habits include limited time spent eating with children (80), frequency of breakfast during the week, the number of eating occasions throughout the day (81), the frequency of family meals (80–82), food choices made within the family (83), inadequate fruit and vegetable consumption (86), and the promotion of a healthy diet as a family (84), eating as an expression of love within the family, daily junk food consumption as a family activity and bonding opportunity (85), using food as a reward (86–88) and eating as a means of controlling the parent–child relationship (86), have been linked to an increase in obesity among children.

There was a significant association between parental obesity or overweight and the prevalence of childhood obesity, as reported in studies (45, 74, 80, 81, 89–94). The link between parental obesity or overweight and child obesity results from both genetic and environmental influences. Family eating habits, together with living conditions and health-related behaviors, determine the weight of children. For instance, overweight or obese parents tend to show decreased attention to their children’s weight problems (95).

According to studies, family income was inversely correlated with being overweight or obese. High rates of overweight and obesity among adults and children were linked to lower income levels (45, 49, 59, 60, 79, 96–98). However, some studies have found a linear positive relationship between family income and BMI (27, 99). Future research should develop longitudinal studies across different regions to track numerous participants because this approach will better understand the causal link between family income and obesity.

The role of economic factors stands essential in creating patterns of obesity beyond socio-cultural influences. The financial situation and food prices together with employment status control both diet choices and physical activity levels. Economic growth, income inequality, unstable economies, and poverty are important factors contributing to the obesity epidemic in the economic category. Studies show that economic growth and rising wealth levels increase overweight and obesity rates in low- and middle-income nations; yet, the connection remains intricate. National GDP levels influence the prevalence of obesity and overweight differently in low-income and high-income countries. The relationship between GDP growth and BMI demonstrates a positive correlation in low-income countries, whereas it exhibits but a negative correlation in high-income countries. (71, 100–103). Other factors such as unemployment (33, 54–56, 67, 77, 104, 105), and urbanization (101, 103), also influence obesity trends. Consumption of poor quality foods containing excess sugar, along with chemical additives associated with poverty, has led to increasing levels of obesity. (72). Factors such as food insecurity (75, 84), low income (32, 50, 64, 67, 79, 106), housing insecurity (67, 107) declining purchasing power (72), and high costs of exercise (108) have been identified as contributors to obesity and overweight, which are closely linked to poverty.

Modern technology has introduced new obstacles to the fight against obesity in the contemporary period. Screen time and food-related digital advertisements create negative impacts on how people conduct their everyday activities. The technology category explores how modern advancements lead to two major effects: simple access to high-calorie food and inactive lifestyles from excessive screen usage and decreased physical movement. Research shows that exposure to unhealthy food marketing through television and online media leads to increased overweight and obesity risks (82, 86, 88, 108–113). Children and teenagers now use modern technology for socializing outside their homes instead of outdoor games and physical activities, so their sedentary engagement with television watching, video games, internet activities and social media has increased. Raising obesity risk for children and adolescents stems from both their sedentary behavior and long periods spent looking at screens. Studies within this age range showed a direct relationship between obesity and lack of sedentary behavior (6, 67, 69, 81, 85, 86, 90, 91, 104, 109, 110, 113–117). Modern distribution systems along with advanced food production methods provide easy access to unhealthy foods that have high energy content in disadvantaged communities (114).

The environmental factors contributing to overweight and obesity include a diverse array of elements within both the built and natural environments that influence individual’s lifestyle behaviors. These factors that affect health outcomes consist of recreational facilities, food access within neighborhoods and climate change and pollution, which directly influence eating patterns and physical activity levels. The environmental category highlighted the significance of the “built environment,” neighborhood’s food environment, pollution and climate, in contributing to obesity. Infrastructure for active living (6, 67, 82, 108), such as suitable sports and recreational facilities (50, 82, 86, 105, 118–121), bike lanes (114), sidewalks (114, 119), green spaces (50, 114, 122), road quality (6), street connectivity (120, 123), and public transport (30, 82, 120, 121, 124), as well as residential density (120) were factors within the “built environment” that effectively address overweight and obesity Another vital aspect in this category is the neighborhood food environment. Factors such as a higher prevalence of convenience stores (40, 125), food availability (28, 82, 86, 88), access to a grocery store (40), unavailability and lack of access to healthy food, availability and easy access to unhealthy foods (67, 68, 83, 85, 109, 112, 113, 125, 126), food environment around schools (127), access to fast-food restaurants (68, 109, 114), lack of healthy choices (83, 105), and access to takeaways (84) were identified as neighborhood food environment factors associated with overweight and obesity.

The political category recognizes that effective policies and regulations play a vital part in tackling obesity on a national scale. A total of six studies explored factors related to political categories. Food and nutrition policies, along with school policies, have been acknowledged as significant factors in the political aspect of obesity. Taxing unhealthy and high-calorie foods (76, 114, 120, 128), subsidizing healthier and less calorie-dense foods (76, 114, 128), implementing food labeling regulations (114), establishing policies for healthy food production and distribution (76), enforcing health regulations on the number, availability, and distribution of high-calorie fast food restaurants (76), promoting the reformulation of processed foods to be healthier (114), setting guidelines for food vendors (89), regulating marketing and advertising, and responding to reduce the impact of advertising on children (86) were identified as factors influencing food and nutrition policies related to overweight and obesity.

Several factors related to school policies that affect overweight and obesity have been recognized. These include implementing physical activity and nutrition guidelines such as conducting simple exercises before class, encouraging walking or cycling to school, eliminating the sale of high-calorie foods, providing healthy food and beverage options, enlisting the assistance of health professionals, and training teachers to utilize the school model BMI (88, 94, 110, 115, 119, 129).

Obesity prevention requires a comprehensive policy approach that encompasses nutritional, behavioral, physical activity, and technological interventions. Strict regulations about unhealthy food advertising should be implemented by governments and public health organizations to protect children from exposure to these unhealthy food advertisements. Government taxation of sugary beverages together with processed foods supports unhealthy food avoidance yet provides financial support for buying nutritious produce including fresh fruits and vegetables and whole grains and lean protein. These measures specifically benefit low-income families in accessing quality food. The prevention of obesity requires equal focus on physical activity promotion. Urban infrastructure expansion through pedestrian-friendly pathways and cycling lanes and green spaces distribution will offer additional exercise possibilities to people. Educational institutions need to create mandatory daily physical education curricula to teach children healthy life practices at their formative stages. Financial benefits that cover gym memberships and weight-loss program discounts would motivate people to integrate physical activity into their daily routine. Employers should support physical activity at work by creating wellness initiatives and establishing standing workstations as well as offering exercise-related rewards to their staff. Lifestyle changes over a long period require behavioral and educational interventions to be successful. The curriculum of schools must incorporate nutrition and health education for teaching students about fundamental balanced diet principles along with physically active living. Behavioral counseling must be accessible to people who experience emotional eating problems and stress-induced eating or struggle with unhealthy food choices. Mental health services through stress management programs and sleep quality improvement and positive self-image promotion can assist obesity prevention goals by minimizing behavioral factors which lead to weight increase. The use of modern digital platforms along with technology-based tools can be helpful in obesity intervention plans. Governments should invest in mobile application development to assist people in monitoring their food intake and exercise activities. The government should establish rules to monitor unhealthy food product advertisements that appear on digital platforms and social media. Additionally, artificial intelligence and big data analytics can be used to monitor obesity trends and design personalized intervention programs based on risk factors in different populations. The interventions need to focus on different population groups for maximum effectiveness. Schoolchildren along with adolescents are among the most vulnerable populations, need protection from unhealthy food marketing and enhanced access to nutritious school meals. Pregnant women and mothers with young children should receive guidance on proper nutrition to prevent childhood obesity from an early stage.

People who are overweight or obese require structured weight-loss programs yet financial incentives help them join lifestyle modification plans. The government should direct subsidies toward low-income communities while expanding healthy grocery options in their residential areas. Workplace policies which promote physical activity while encouraging healthier eating habits will enable employees and workers to become active participants in obesity prevention.

The achievement of obesity prevention policies requires multiple stakeholders to work together. Governments and public health policymakers must take the lead in regulating, allocating resources, and designing national health programs. Healthcare providers who include doctors and nutritionists and mental health professionals should provide counseling and medical care to individuals who risk becoming obese. Schools and educational institutions must ensure that students have access to the necessary education on nutritious food and physical education. The food and beverage industry needs support to reformulate products containing less sugar and fat while practicing responsible marketing methods. Media platforms need to limit unhealthy food advertising while they should promote healthy life benefits. Employers should establish workplace health plans, and non-profit organizations, along with local communities, can play an important role in raising awareness and supporting at-risk populations.

Obesity prevention programs will become more effective and sustainable when these strategies are implemented at various levels starting from government regulations down to community-based programs. The implementation of a multisectoral coordinated strategy reduces obesity rates and overweight while simultaneously improving public health results and decreasing health care expenses and boosting overall life quality.

### 4.1 Strengths and limitations

The scoping review examines multiple factors which influence overweight and obesity across socio-cultural, technological, economic, environmental and political domains. The main research strength emerges from its transparent methodological procedures and rigorous approach. The research design followed the frameworks provided by Arksey and O’Malley and JBI guidelines to execute systematic identification and synthesis of relevant literature. A combination of quantitative and qualitative research with different databases increases the reliability and generalizability of the results discovered. Additionally, the thematic synthesis approach provides an in-depth evaluation of how various significant factors associate with one another which generates essential information for designing public health interventions and policies.

Several limitations need attention when considering this information. The methodology of scoping reviews does not involve a quality assessment of the included studies, so this could introduce variability in the reliability of the findings. Additionally, there is an inherent limitation in the geographical distribution of the included studies. Research mainly took place in high-income countries throughout North America and Europe without sufficient investigation in low-income nations. The limited participation of low-income countries in the research sample accounts for only 5% while this underrepresentation could affect the applicability of study results to those specific regions. Moreover, the unequal geographical distribution reveals an existing knowledge gap in academic research about the special socio-economic and environmental difficulties experienced by low-income populations. Additional research should focus on the neglected areas because it will help develop an inclusive understanding of the worldwide obesity crisis.

## 5 Conclusion

Research studies on obesity and overweight distribution show increasing prevalence worldwide but these studies remain scarce in low- and lower-middle-income countries. The majority of studies in the literature include cross-sectional analyses, but longitudinal surveys combined with qualitative and mixed methods help to understand the complex nature of obesity. The trends of obesity demonstrate strong links to socio-demographic factors which include age, gender, marital status, education and socioeconomic status. Obesity rates are higher among younger age groups even though older people tend to have higher BMI values which emphasizes the need for prevention programs at an early age. Men tend to be overweight more often than women yet women face greater obesity challenges because of their inactive lifestyles and sociocultural norms. Marital status impacts weight gain differently since married people and those who are divorced or widowed experience more obesity risks because of their altered lifestyles. Education and economic status play crucial roles in dietary choices and physical activity levels, though their impacts vary across different demographic groups. Rural areas are now facing increasing obesity rates despite their historical association with lower obesity levels due to changes in food environments and reduced physical activity. Early-life interventions must focus on families because their education level and occupation status, income, and eating habits, along with their financial situation and dietary practices directly affect childhood obesity rates. The economic factors including GDP growth, unemployment and food insecurity affects obesity occurrence particularly within low- and middle-income nations. Modern technological developments intensify obesity patterns because they lead to higher screen usage and food marketing and less physical activity particularly impacting young people. Environmental factors including built environments, food accessibility as well as climate-related determine obesity rates because they influence lifestyle behaviors. Finally, the prevention of obesity benefits from political interventions including food taxation in addition to subsidies for nutritious choices and requirements for food labeling and physical activity education in schools.

The worldwide goal to lower obesity levels faces multiple connected obstacles making this health issue especially challenging to tackle. Cheap and readily available unhealthy food products currently control the market with their high energy density, and advertisers specifically target children (130, 131). The lack of safe places for physical exercise combined with declining active lifestyles that substitute walking with driving creates a more severe problem in urban settings (128, 129). Additionally, the higher prices of nutritious foods restrict access for numerous people including low-income groups at high risk of obesity (132). Healthcare systems struggle with funding obesity-related diseases which hinders their ability to establish preventive measures (133, 134). The experience of social stigma stops many individuals from getting assistance because it produces feelings both of shame and low self-esteem (135, 136). The normal acceptance of obesity within certain cultures presents an additional challenge when trying to change social perspectives about weight health (135). Despite numerous prevention programs, many face a major challenge since they approach obesity solely through diet or physical activity while failing to combine these strategies (7, 136). Despite the implementation of policies such as food labeling and taxes on sugary drinks, their success is often undermined by inconsistent implementation and a lack of public support in the long term (137, 138). Lastly, individuals maintain overeating patterns because of behavioral triggers while their psychological factors include emotional eating and the comfort of high-calorie foods (139, 140). The strong relation between emotions and psychology and food consumption demonstrates why comprehensive weight management solutions must target bodily needs as well as emotional aspects of obesity.

This study highlights the multifactorial nature of obesity and the complex interactions among its numerous contributing factors. A total of 82 factors influencing overweight and obesity were identified and categorized using the STEEP framework into five major groups: sociocultural, economic, technological, environmental, and political. The sociocultural category, as one of the most important determinants of overweight and obesity, included the highest number of influencing factors. This highlights the significant impact that family structure, social support, and cultural norms have on health behaviors. Environmental factors along with technological developments combined with economic circumstances have strongly influenced the increasing incidence of obesity. Concurrently, political influence such as public health policies together with regulatory frameworks operate as political factors that are essential in establishing the broader framework for obesity prevention initiatives.

A holistic comprehensive strategy needs to address overweight and obesity issues stemming from multiple influencing factors. Such an approach requires coordinating evidence-based interventions from numerous sectors to tackle all factors that affect obesity at individual and different societal levels and create sustainable change. The approach requires multi-sectoral interventions that analyze the various sociocultural along with economic and environmental and political dynamics across essential domains such as health, education, transport, agriculture and the environment. The strategy needs to include all age groups and social classes but specifically target vulnerable population groups. The approach should merge educational programs for individual health with environmental changes that promote healthier decisions. A meaningful outcome requires collaboration between stakeholders including government, private sector, food industry and educators, planners, schools, local communities and media. Lastly, it is important to continue evaluation process through policy monitoring together with data-based decision-making for adjusting strategies.

## Data Availability

The original contributions presented in the study are included in the article/Supplementary material, further inquiries can be directed to the corresponding author.
