# Supplementary material for: Exploring the multifaceted factors influencing overweight and obesity: a scoping review
Source: Front Public Health. 2025 Apr 9;13:1540756. doi: 10.3389/fpubh.2025.1540756 (PMC12014677; doi:10.3389/fpubh.2025.1540756)
Supplement: Supplementary file 1 [file Table_1.docx]

| **Title of the study** | **Author and date** | **Region** | **population/**  **participants** | **age group** | **Study design** | **Aim of the study** | **key factor related to OW/OB** |
| --- | --- | --- | --- | --- | --- | --- | --- |
| Overweight and obesity and its socio-demographic correlates | Abrha S, et al. 2016 | Ethiopia | women  age 15-49 (n=3,602) | adult | cross-sectional | assessing the prevalence and socio-demographic correlates of overweight /obesity | Being married, older, belonging to the richest quintile, educational level, living in the metropolises |
| Cultural factors related to childhood and adolescent obesity | Aceves-Martins M, et al .2022 | Mexico | 24 studies | child and adolescent | review | identify decisions, behaviors, individual experiences, perceptions, attitudes, or views related to obesity | conception of a good diet , food marketing campaigns, food customs, food availability and taste, obesity perceived as an esthetic issue not a health problem, sugary drinks, children having money during school hours, cooking methods , Rewarding children with food, food as controlling the parents' and children's relationship, Sedentary lifestyles, Beliefs about health, Eating rituals, Physical activity , Dietary habits, lack of time of some working parents, Employment of mothers, expensive nutritious food, lack of suitable spaces or materials for physical or recreational activities, Access to computers/internet, social media, communication, lack of policies to regulate food vendors, lack of strategies and guidelines to encourage physical activity, don’t access to education |
| Trends and determinants of underweight and overweight/obesity | Ahmed KY, et al. 2020 | Ethiopia | women age 15–49  (n=10,346) | adult | cross-sectional | investigate association between socioeconomic, demographic, behavioral, and community-level factors with overweight and obesity | wealthier households, education, watching TV, Informal employment and listening to the radio |
| Prenatal traffic-related air pollution exposures and infant weight | Alderete TL, et al. 2018 | USA | mother-infant pairs (N=136) | child | longitudinal | examine associations between traffic-related air pollution exposure and infant weight change | air pollution |
| Disparities in Obesity Prevalence in Adults | Amin R, et al. 2021 | Iran | adults aged ≥18 (n=28,321) | adult | cross-sectional | outlines the social determinants of obesity | gender, age, married, living in urban setting, occupational status, marital status |
| Global warming and obesity | An R, et al. 2018 | USA | 50 studies | NA | review | findings on the relationship between global warming and the obesity epidemic | global warming |
| Sex difference in the association of obesity with personal or social background | Asahara S, et al. 2020 | Japan | adults age 20-64 (n=5425) | adult | cross-sectional | gain insight into environmental factors that contribute to obesity | unmarried status, a low household income, welfare enrollment, difficult current economic conditions, a low educational level, and childhood adversity |
| Socio-demographic predictors of obesity | Athieno J, et al. 2023 | Uganda | women age 18-69 (n=384) | adult | cross-sectional | exploring social and demographic predictors of obesity | Age, marital status, childbearing status, employment status |
| Spatiotemporal distribution and determinants of overweight or obesity | Azanaw MM, et al. 2022 | Ethiopia | women age 25-49  (n=7,752) | adult | cross-sectional | determining determinants of changes in overweight/obesity over time | age, educational status, marital status, and employment status |
| Overweight and obesity among adults in the Gulf States | Balhareth A, et al. 2019 | Gulf States | 91 studies | adolescent and adult | review | explore the scientific evidence on correlates and interventions for overweight | Low physical activity, sedentary behavior, unhealthy dietary habits, age, being married, low education, urban residence, and unemployment |
| Determinants of Weight-Related Behaviors | Balhareth A, et al. 2021 | Saudi Arabia | students age 20- 22 (n=33) | adult | qualitative | explore the potential determinants of weight change | eating behaviors, physical activity, sedentary behaviors, inadequate sleep, lack of knowledge and time and stress, cultural aspects, weather conditions, passive transport dependency and khat consumption |
| Using photovoice to explore social determinants of obesity | Bateman LB, et al. 2019 | USA | adults age ≥21 (n=10) | adult | qualitative | explore the social determinants and examine residents’ perceptions about contributors of obesity | lack of healthy choices, depression, poverty, security, Community (economic) development and employment opportunities, strategic placement and display of foods, Recreational environment, Social cohesion, civic engagement, collective efficacy, working long hours at more than one job |
| Rising rural body-mass index is the main driver of the global obesity epidemic | Bixby H, et al. 2019 | International  (190 country) | adults age ≥ 18  (n=112 million) | adult | cross-sectional | report national, regional and global trends in mean BMI segregated by place of residence (a rural or urban area) | rural areas |
| Assessment of sleep and obesity | Bonanno L, et al. 2019 | Italy | 71 adults age 29 -65, 128 children age 10-13 | adolescent and adult | cross-sectional | investigated the association between sleep duration and quality, and overweight risk and obesity | Quantity and quality sleep |
| Modifiable Determinants of Obesity in Native Hawaiian and Pacific Islander Youth | Braden KW, Nigg CR. 2016 | USA | 14 studies | child and adolescent | review | assess modifiable determinants or correlates of overweight and obesity | infant-feeding mode, geographic location, parental education |
| Association Between Neighborhood Factors and Adult Obesity | Brakefield WS, et al .2022 | USA | NA | NA | cross-sectional | investigate the effects of social determinants of health on obesity | median household income, home renters, living below the poverty level, 55 years or older, unmarried, and uninsured, black race |
| Adolescent obesity in the past decade | Campbell ET, et al. 2019 | USA | 41 studies | adolescent | review | appraise the state of food choice and genetic determinants of adolescent overweight/obesity | commercial competition from the fast food and convenience food, financial independence, taste, ethnic/family culture, family structure, prevalence of more convenience stores, nuclear and blended family, genetic, Cultural beliefs, values, transition to the higher processed foods, snack foods and beverages, Stress, education |
| An obesogenic island in the Mediterranean: mapping potential drivers of obesity | Cauchi D, et al. 2015 | Malta | 109 studies | all | review | explore factors that may have potentially contributed to the establishment of an obesogenic environment | infrastructure for active living , energy-dense food supply, food availability, domestic expenditure decreases, food pricing, unhealthy snack, availability and taste, lifestyle and socio-economic status, access to sports and recreational facilities, public transport, sustainable consumption of fruit and vegetables, online media advertising, working mothers, time spent watching TV, playing video games, eating meals together as a family, National Obesity Strategy, policy response to reduce the impact of advertising on children |
| Conceptualizing the commercial determinants of dietary behaviors associated with obesity | Chavez-Ugalde Y, et al. 2021 | international | 81 studies | adolescent | review | providing commercial determinants of dietary behavior associated with obesity | influencing policymaking processes through lobbying, influencing governance of food production, trade, and investment by taking advantage of neoliberal economic policies, marketing and preference |
| Socio-Demographic, Behavioral and Psychological Factors Associated with High BMI | Chew HSJ, et al. 2023 | Singapore | age ≥21 (n = 247) | adult | cross-sectional | examining the relationship between socio-demographic, behavioral and psychological factors with overweight and obesity | race, education, marital status |
| Prevalence and Predictors of Obesity among School children | Chomba H, et al. 2019 | Tanzania | schoolchildren age 7-17 (n=451) | adolescent | cross-sectional | investigate the prevalence and predictors of childhood obesity | gender, random sleeping time and random eating habit |
| Influencing Factors of the BMI of Elementary Students | Chou LN, Chen ML. 2017 | Taiwan | students age 9-10 (n=3,251) | child | cross-sectional | investigate the influencing factors and perceived body shape on the BMI | gender, physical activity levels, sedentary behaviors, diet habits, and body shape satisfaction |
| The social determinants of health influencing obesity for the aged | Chumpunuch P, Jaraeprapal U, 2022 | Thailand | stakeholders (n=19) | adult | qualitative | describe the social determinants of health influencing obesity for the aged | easy access to unhealthy food, no choice to recruit healthy food, family affects food choices, prohibitions on exercise; belief, socially imposed body image perceptions, lack of awareness, personal attitudes, job and familial duties, over-consumption behaviors |
| A foresight whole systems obesity classification for the English UK biobank cohort | Clark S, et al. 2022 | UK | adults age 40-69 year (n = 345,091) | adult | qualitative | understanding the collective characteristics and behaviors of those who are overweight or have obesity | unemployment, watch TV, consumption of vegetables and fruit, stressful events, number of years that they have been a smoker, satisfaction with health, levels of neighborhoods deprivation |
| Food prices, taxes, and obesity and its implications for food taxation | Clark SJ, et al. 2018 | Canada | adult age ≥18 | adult | cross-sectional | highlights the importance of addressing obesity by considering nutrient prices in studies and policy-making, rather than focusing on food group prices. | taxing fat and carbohydrates, subsidizing protein |
| Obesity trends and risk factors in the South African adult population | Cois A, Day C .2015 | South Africa | adults age≥ 18 (n=10,100) | adult | longitudinal | identification of risk factors for progression towards obesity | socioeconomic status, gender, age, race, household income per capita, quitting smoking, rural dwellers, physical inactivity |
| Ethnicity, socioeconomic status, and overweight in Asian American adolescents | Cook WK, et al. 2016 | USA | adolescents age 12–17 (n=1,533) | adolescent | cross-sectional | identify specific profiles of Asian subgroups at high risk of adolescent overweight | family incomes, ethnic-group, SES |
| Prevalence of overweight and obesity and associated factors | da Silva AP, et al. 2018 | Brazil | children and adolescent’s age  5-18   (n=1,125) | child and adolescent | cross-sectional | describe the prevalence of overweight and obesity and associated factors | time spent watching TV or participating in media-related activities ≥ 5 hrs/day, higher economic class and education level of head of the family (≥12 years). |
| The epidemiological burden of obesity in childhood | Di Cesare M, et al. 2019 | international | children age 2-4 and age 5-19 | child and adolescent | cross-sectional | provide a comprehensive, reliable and detailed estimates of the worldwide epidemic of excess weight in children and adolescents | availability of healthy foods and unhealthy options, marketing, tax on beverages containing sugar, subsidies for producing and distributing fruit and vegetables, regulations on food labelling, advertising, incentives or regulations to catalyze reformulation of processed foods toward healthier composition |
| Sleep and Obesity | Ding CZ, et al. 2018 | USA | 50 studies | adult | review | summarizes the most recent evidence linking decreased sleep duration and poor sleep quality to obesity | sleep duration, sleep disturbance, sleep quality |
| Disentangling the Drivers of Obesity | Dogbe W, et al. 2021 | Spain | households (n=180) | adult | cross-sectional | investigate how intrapersonal and socioeconomic factors affect body weights | income, marital status, gender, age, attitude toward obesity, weight stigma, belief about the controllability of obesity, correct weight perception, risk attitudes and loss aversion |
| Childhood predictors of adult obesity | Eales L, et al. 2020 | USA | child (n=1,065) | child | longitudinal | identify childhood predictors of adult obesity | socioemotional learning, neighborhood human capital, gender, higher birth weight, family history of respiratory diseases, childhood asthma |
| Obesity and overweight: prevalence and associated socio demographic factors | El Keshawi RR, et al. 2014 | Palestine | mothers age 18-50 (n=357) | adult | cross-sectional | determine obesity and overweight prevalence and the associated factors. | increasing age, family monthly income, housewives |
| The Complex Etiology of Childhood Obesity in Arabs | Elkum N, et al. 2019 | Kuwait | schoolchildren age 6-18 (n=6,574) | child and adolescent | cross-sectional | identify predictors of childhood and adolescent obesity | birth weight, maternal employment, maternal age at pregnancy, family size, gender, age |
| Food reinforcement and parental obesity predict future weight gain | Epstein LH, et al. 2014 | USA | 130 non-obese adolescents | child | longitudinal | assess the independent effects of food reinforcement and parental obesity on weight gain for adolescents | Food reinforcement and parental obesity |
| Hierarchical analysis of dietary, lifestyle and family environment risk factors for childhood obesity | Farajian P, et al. 2014 | Greece | children age 10-12 (n=4,552) and parents (n=2,225) | child | cross-sectional | recognize the most important factors that are associated with childhood overweight/obesity | breakfast frequency, daily number of meals and snacks, the frequency of family meals, having both a TV and a PC/video game player in the bedroom, study hours on weekdays, mothers' age, maternal and paternal BMI and children's BMI misclassification |
| Obesity in Low- and Middle-Income Countries: Burden, Drivers, and Emerging Challenges | Ford ND, et al. 2017 | low and middle-income country | NA | all | review | review the distinctive features of excess weight | gender, urban settings, age, global diet, physical activity, food availability and pricing, globalization, trade liberalization, Sleep deprivation, environmental contaminants, chronic psychosocial stress, neuroendocrine dysregulation, and genetic/epigenetic mechanisms |
| What is driving global obesity trends? Globalization or "modernization"? | Fox A, et al. 2019 | International  (190 countries) | NA | NA | cross-sectional | evaluate the influence of economic globalization versus economic development and associated processes on global weight gain | GDP per capita, urbanization, women's empowerment, economic growth |
| Trends in the Association of Parental History of Obesity | Fox CS, et al. 2014 | USA | adults age 28-62 (n= 5,209), Offspring and their spouses (n=5,124), Third Generation (n=4,095) | adult | longitudinal | the association of familial as compared to genetic factors in the current obesogenic environment | parental obesity |
| Relationship between childhood obesity and socio-economic status | Gamboa-Gamboa T, et al .2021 | Costa Rica | children age 6-12 (n=347366) | child | cross-sectional | analyses the relationship between socio-economic status and the prevalence of overweight and obesity | drinks at tuck shop, availability of policy on physical activity and training teacher as a role |
| Ranked Importance of Childhood Obesity Determinants | Glover M, et al. 2019 | New Zealand | adults aged ≥ 16 (n=180) | adolescent and adult | qualitative | explore the relative influence of factors contributing to body weight in children | cost of healthy foods, access to takeaways, lack of time for food preparation, screen time, lack of time to ensure children exercised, lack of familial, social and health promotion support, family support, not access to culturally appropriate nutrition education or social support and services, economic deprivation on food insecurity |
| Social determinants of obesity in American Indian and Alaska Native peoples | Goins RT, et al. 2022 | USA | age ≥ 50 (n=27696) | adult | cross-sectional | assessing social determinants of obesity | age, gender, don’t having Medicaid coverage, private health insurance, living in areas with lower rates of educational attainment and longer drive times to primary care services, access to a grocery store |
| Economic development, urbanization, technological change and overweight | Goryakin Y, Suhrcke M. 2014 | International (56 countries) | non-pregnant women age 15–49 (n=878,000) | adult | cross-sectional | Examining the role of various indicators of economic development, urbanization and technological changes in explaining overweight | Shifting patterns of employment from agriculture into services, Urban residence, SES, GDP per capita, car and TV ownership, Greater per capita calorie intake, education, economic shock, national per capita income |
| Research on Environmental Influencing Factors of Overweight and Obesity | Guo YR, et al. 2022 | China | children and adolescents age 10–18 (n=26120) | child and adolescent | cross-sectional | exploring the impact of environmental factors on overweight and obesity | latitude, altitude, family SES level, gross domestic product (GDP), and level of urbanization |
| Socioeconomic and gender inequalities in childhood obesity | Gutiérrez-González E, et al. 2023 | Spain | children age 6-9 (n=16,665) | child | cross-sectional | exploring the association between childhood weight status and household socioeconomic status | low socioeconomic status, household income, parental education, parental employment status |
| Neighborhood and Individual Sociodemographic Characteristics Associated with Disparities | Halpern MT, et al. 2017 | USA | adults ≥18 (n=21,531) | adult | cross-sectional | assess associations among overweight and obesity with neighborhood and individual sociodemographic factors | age, gender, race/ethnicity, household income, education |
| Nontraditional risk factors for obesity in modern society | Han SJ, Lee S. 2021 | south korea | NA | all | meta-analysis | reviewe novel environmental factors in modern society in obesity | light pollution, air pollution, endocrine-disrupting chemicals, reduced greenspace |
| Trends and Factors Associated with Obesity Prevalence | Hannah S, et al. 2022 | AUS | adult age ≥18 (n = 7907) | adult | cross-sectional | examining the changes in the prevalence of obesity and associated lifestyle factors | fried food, fruit intake, older age (≥35 years), use of fat-based spreads for breads, physical inactivity, food insecurity, higher price of healthy foods, rural towns, physical inactivity |
| Association between Obesity and History of Abuse | Hodge F, et al. 2014 | USA | age ≥18 (n=459) | adult | cross-sectional | explore factors associated with obesity among American Indians | history of high blood pressure and diabetes, having a history of verbal abuse in childhood |
| Socioeconomic Inequalities in the Rise of Adult Obesity | Hoebel J, et al. 2019 | Germany | Aadults age 25-69 (n = 18,541) | adult | cross-sectional | examined secular trends in obesity prevalence by socioeconomic position and the resulting obesity inequalities | socioeconomic groups, income, education |
| The association between living environmental factors and adolescents' body weight | Huang SY, et al. 2021 | China | children age 11-15 (n=2188) | adolescent | cross-sectional | explore the relationship between body weight and environmental factors | the number of sports venues and bus stops in the neighborhood |
| An exploration of the determinants of overweight and obesity and the capacity to intervene | Hughes R, et al. 2023 | AUS | stakeholders (n=35) | NA | qualitative | identify determinants, needs, strategic priorities and capacity to act on overweight and obesity prevention | unhealthy eating , inactive lifestyles, Community connections, harsh winter climate, Access to PA infrastructure and services, socializing opportunities, housing insecurity, income, unemployment, employment loss, low levels of educational attainment, the unavailability of cheap, healthy food, not working , watching TV all day, drinking alcohol, mental health |
| Effects of school neighborhood food environments on childhood obesity | Jia P, et al. 2019 | USA | kindergarteners (n=7,530) | child | longitudinal | examine association between School neighborhood food environment and childhood obesity | food environment around schools |
| Natural environment and childhood obesity | Jia P, et al. 2021 | international | 6 studies | child and adolescent | review | reviewe associations between a full range of natural environmental factors and obesity | altitude, weather temperature |
| Environmental determinants of childhood obesity | Jia P, et al. 2023 | international | 457 studies | child | meta-analysis | translate existing obesogenic environmental studies into evidence-based governance for fighting childhood obesity and promoting life-course health | access to fast-food restaurants, fast-food consumption, access to bike lanes, access to sidewalks, sedentary behaviors, access to green space, TV or computer screen time |
| the Prevalence and Determinants of PRECEDE Framework and Health Literacy Associated with Overweight and Obesity | Kaewchin P, Banchonhattakit P. 2019 | Thailand | adolescents age 12-19 (n=1,129) | adolescent | cross-sectional | investigate the prevalence and determinants of PRECEDE Framework and health literacy associated with overweight and obesity | low levels of attitude and reinforcing factors, low levels of health literacy including media literacy skills, decision skills, watching television ≥3 hours per day, watching TV while eating, time to surf internet >5 hours per day |
| Childhood overweight and obesity and associated factors | Khashayar P, et al. 2018 | Iran | students and their parents (n=14,880) | child and adolescent | cross-sectional | explore multidimensional factors related to childhood obesity and overweight | living in urban area, increasing age, high and moderate SES, high BW and family history of obesity, breakfast skipping |
| Update on the Obesity Epidemic: Is the Sharp Rise of the Evil Empire Truly Levelling Off? | Koliaki C, et al. 2023 | international | 21 studies | all | review | exploring the underlying drivers of the global obesity epidemic | international food production and supply system, local environmental factors (fast food restaurants, supermarkets, parks, transportation facilities) ,Improved food manufacturing and distribution systems and pervasive marketing campaigns, widely accessible unhealthy and energy-dense foods, access to cheap /palatable/highly processed of minimal nutritional quality, obesogenic chemicals with endocrine-disrupting properties , changes in the dietary composition, physical activity , alterations in the gut microbiome, television and computer devices , increased energy intake |
| Longitudinal Trends in Body Mass Index Before and During the COVID-19 Pandemic | Lange SJ, et al. 2021 | USA | age 2-19 (n=432,302) | child and adolescent | longitudinal | examine differences in the average rate of change in BMI before and during the COVID-19 pandemic | covid 19, public health emergencies |
| Identifying Key Determinants of Childhood Obesity | LeCroy MN, et al. 2021 | international | 15 studies | child and adolescent | review | summarize the key determinants of obesity identified in existing machine learning studies | child's weight history, parental overweight/obesity, maternal weight history, , learning English as a second language in school, more teachers per student , financial independence, weight perception and acculturative stress |
| Perceived environmental factors associated with obesity | Lemamsha H, et al. 2018 | Libya | adults age 20-65 (n=401) | adult | cross-sectional | examine associations between perceived neighbourhood built environmental attributes and obesity | street connectivity, unsafe environment, committing crimes at night, neighbourhood aesthetics, access to public transport, access to recreational facilities, residential density zones |
| Using Social Media to Understand Web-Based Social Factors Concerning Obesity | Li C, et al. 2022 | USA | 50 studies | all | review | examining web-based social factors in relation to obesity research | social support, gender, source credibility, social movements, social sharing behaviors, stigma, policy, school environment, geo-cultural factors, obesogenic environment |
| Associations of Diet Quality and Heavy Metals with Obesity in Adults | Li TZ, et al. 2022 | China | age ≥ 20 (n=15959) | adult | cross-sectional | exploring the associations between diet quality and heavy metals and obesity | heavy metals, lower diet quality |
| The trajectory and the related physical and social determinants of body mass index | Lin LJ, et al. 2014 | Taiwan | children age 7 (n=1609) | child | longitudinal | explore developmental trajectory patterns of BMI and associated factors | after-school exercise, academic performance, family interactions, overweight parents, father's education level, television viewing or computer use, peer interaction |
| Social determinants and behaviors associated with overweight and obesity | Macicame I, et al. 2021 | Mozambique | adults age 15-64 (n=843) | adult | cross-sectional | assess social and behavioral determinants of obesity | gender, age, living in a wealthier household, low level of education |
| Social Inequalities in Obesity Persist in the Nordic Region Despite Its Relative Affluence and Equity | Magnusson M, et al. 2014 | The Nordic countries (Sweden, Denmark, Finland, Norway and Iceland) | children age 2-9 and middle age 25-64, elderly residents | all | review | assess the presence of social inequality in overweight /obesity | social inequality, low parental education, income levels, parental occupational status, rural area |
| Dietary patterns and child, parental, and societal factors associated with being overweight and obesity | Mai TMT, et al. 2023 | Vietnam | children age 9–11 (n=221) | child | cross-sectional | assess child characteristics, dietary patterns, parental and societal factors for associations with childhood overweight and obesity status | dietary patterns with discretionary (snacks and sweetened beverages), gender, screen time over 2 h/day, parental underestimation of child weight status, father's obesity, and household income in the lowest quintile |
| Assessing risk factors that can cause overweight and obesity | Makarova EL, et al. 2020 | Russia | women age 15–49  (n=226) | adult | cross-sectional | examine certain risk factors and assess their effects on overweight /obesity | unhealthy nutrition, physical activity, age being 35 and older, smoking , gastric and liver disease, sleep disorders |
| The built environment as determinant of childhood obesity | Malacarne D, et al .2022 | UK | 14 studies | Child and adolescent | review | evaluation the epidemiological evidence on the built environment and its link to childhood obesity | traffic-related air pollution, street intersection, not access to parks |
| Societal risk factors for overweight and obesity | Mangemba NT, San Sebastian M. 2020 | Zimbabwe | adult female age 15-49  (n = 8904) | adult | cross-sectional | determine the socioeconomic risk factors for overweight and obesity | older age, being married, being wealthy, use of hormonal contraception, higher education, religion |
| Analysis of main risk factors contributing to obesity | Marincová L, et al. 2020 | East Africa countries | 16 studies | all | Meta-analysis | analyse the predictors of obesity | gender, type of residence, socioeconomic status |
| Factors influencing obesogenic dietary intake | Mazarello Paes V, et al. 2015 | USA, Europe and Australia | 22 studies 1,067 participants | child | review | describe the barriers to and facilitators of obesogenic dietary intake in early childhood | negative parent/family/peer modelling, lack of knowledge, time constraints, using food as reward, affordability, concerns about child's health. Child preferences, availability, advertising, societal, cultural and preschool/childcare influences |
| Neighborhood Social Predictors of Weight-related Measures | McDaniel TC, et al. 2015 | USA | adults age >18  (n=417) | adult | cross-sectional | examine whether factors based on a bioecological framework contribute to predicting weight-related measures | neighborhood social interaction |
| Comparing complex perspectives on obesity drivers | McGlashan J, et al. 2018 | AUS | stackholder (n=50) | NA | qualitative | present comparison between the Foresight obesity systems map and a community-developed map of the drivers of obesity | marketing and advertising, perceived lack of time, physical activity, local infrastructure, school policy, cost of exercise |
| Demographic, socio-economic and behavioral correlates of BMI | Micklesfield LK, et al .2018 | South Africa | adults age 44–54 (n=2035) | adult | cross-sectional | identify the demographic, socio-economic and behavioral factors associated with BMI | age, gender, marital status, household asset score |
| The Association Between Perceived Discrimination and BMI Trajectory | Miller HN, et al. 2022 | USA | adults age 30-64 year  (n=1,962) | adult | longitudinal | examining the association between perceived discrimination and BMI trajectory | perceived discrimination |
| Prevalence and factors associated with overweight and obesity in Kenya | Mkuu R, et al. 2021 | Kenya | adults aged 18-69 (n=4,340) | adult | cross-sectional | examined the prevalence, sociodemographic, and behavioral risk factors associated with having overweight /obesity | married individuals, gender, urban dwellers, middle wealth or higher, age, education |
| Air pollution and childhood obesity | Moon Young S, et al. 2020 | international | 38 studies | child and adolescent | review | review updated information on air pollution in childhood obesity | air pollution |
| Global human obesity and global social index: Relationship and clustering | Munir M, et al. 2023 | International (183 countries) | NA | NA | review | Determining the role of socialization, as one of the important dimensions of overall globalization, in increasing the risk of overweight and obesity | political globalization, quality of Infrastructure and institution, human development index, social globalization, global happiness, internet usage and quality of roads |
| Why are primary school children overweight and obese? | Mwaikambo SA, et al. 2015 | Tanzania | children age 7–14 (n=1,722) | child and adolescent | cross-sectional | determine the prevalence and factors associated with overweight /obesity | walked to and from school, private cars or school buses use, Computer/video game use, private schools, Lunch provided by schools |
| Prevalence of Obesity and Abdominal Obesity and Social Factors | Nam GE, et al. 2020 | south korea | all age (n=NA) | all | cross-sectional | addressed the prevalence of obesity and obesity-related social factors | age, gender, lower levels of education and household income, managers, working more hours per week, split-shift and night-shift workers |
| Socioeconomic inequalities in intergenerational overweight and obesity transmission | Nglazi MD, Ataguba JE. 2022 | South Africa | non-pregnant mothers age 15 -49 (n=12,157) and offsprings (n= 15,014) | adolescent and adult | cross-sectional | estimate and decompose the socioeconomic inequality in the intergenerational transmission of overweight and obesity from mothers to offsprings | wealthier mother-offspring pairs, mothers' education, socioeconomic status, household size, employment status, urban residence |
| Family-Related Characteristics and Childhood Obesity | Notara VMP, et al. 2020 | international | 58 studies | child and adolescent | review | examine association between family factors as well as family structure and childhood overweight /obesity | parental weight, parental educational status, parental occupational status (high occupational level, two out-of-home working parents), family structure, family meals frequency, parenting styles, feeding practices, family perception about child’s weight and family history of diseases |
| Effect of the COVID-19 pandemic on obesity and its risk factors | Nour TY, AltintaŞ KH. 2023 | International (22 countries) | 40 studies | all | review | investigate and determine the determinants of obesity during the coronavirus disease (COVID-19) pandemic | covid 19, physical inactivity, sedentary lifestyle, poor eating patterns, unhealthy eating habits, excessive behavioral stress, depression, anxiety, low mood, age, gender, and ethnic minorities |
| Overweight in Indonesia: an observational study of trends and risk factors | Oddo VM, et al. 2019 | Indonesia | children age 0–18 and adults ≥19 | all | longitudinal | assess associations between key hypothesized determinants and overweight | urban area residence, wealth, education, consumption of ultra-processed foods |
| Machine learning approaches to characterize the obesogenic urban exposome | Ohanyan H, et al. 2022 | Netherland | adults age 31-65 (n=14829) | adult | cross-sectional | exploring what environmental factors of the urban exposome are related to BMI | average neighborhood value of the homes, air pollution (OP), healthy food outlets in the neighborhood (5 km buffer), one-person households in the neighborhood, lower average house values, lower share of one-person households |
| Association of socioeconomic position and childhood obesity | Paalanen L, et al. 2022 | Finland | Children aged 2-17 (n=194,423) | child and adolescent | cross-sectional | identify what dimensions of socioeconomic position (SEP) are most closely associated with childhood obesity | annual household income, mother and father's educational level, The parents' SEP |
| Economic Determinants of Obesity and Overweight in Ten Post-Communist CEE Countries – Similar Trends? | Pană MC, et al. 2022 | Bulgaria, Czech Republic, Estonia, Hungary, Latvia, Lithuania, Poland, Romania, Slovakia, Slovenia. | Children age 5-9 Adolescents 10-17 Adults ≥18 | ALL | cross-sectional | Showing the effect of economic, social and demographic factors on obesity and overweight | poverty, stronger propensity for low-quality sugar-dense food with chemical additives, low incomes, decreasing purchasing power, price index, consume lower quality food, cheaper products, blood glucose, high education, migration, |
| Economic growth as an underlying probable systemic driver for childhood obesity | Pisa PT, et al. 2021 | South Africa | youths (<24 years) (n= 13,341) | adolescent and adult | cross-sectional | demonstrate childhood obesity trends and explore their associations with economic growth | GDP per capita, Gini coefficient |
| Overweight and obesity in childhood and adolescence | Pongiglione B, et al. 2019 | UK | 9 months to 14 (n=10,825) | child | cross-sectional | Identify factors associated with the risk of overweight /obesity | maternal education, breastfeeding, home ownership, child’s active behavior, puberty, birth weight, sleeping behavior, smoking mother during pregnancy |
| Sociodemographic disparities and contextual factors in obesity | Pou SA, et al. 2022 | Argentina | adults age ≥ 18 (n=16410) | adult | Cross-sectional | assessing the association of sociodemographic and environmental factors with the obesity | gender, age, being married, living in the city, lower education, low-income level, multi-person household, availability of socially constructed recreational resources and green spaces |
| The socio-economic inequality in body mass index | Pourfarzi F, et al. 2022 | Iran | Adults age 35-70 (n = 20,460) | adult | Cross-sectional | exploring and determine the association between BMI and socio-economic factors | age, gender, married, lower education level, having chronic disease, alcohol use, richest people |
| Relationship between perceptions about neighborhood environment and prevalent obesity | Powell-Wiley TM, et al. 2013 | USA | Adults age 18-65 ( n= 5,907) | adult | cross-sectional | examine the association between perceptions of neighborhood environment and obesity | heavy traffic, trash/litter in neighborhood, lack of recreational areas, lack of sidewalks |
| Use Factor Analysis in Determining Most Important Factors Affecting Childhood Obesity | Qadouri AS, et al. 2021 | Iraq | children age 5-12  (n=150) | child and adolescent | cross-sectional | identifying the most important factors causing obesity | marital status of mother, position of the child within family, family's economic situation, work of mother, time to sleep, possession of a mobile phone,   consumption of sweetened juices and soft drinks |
| The Predictors of Obesity among Urban Girls and Boys | Ratajczak J, Petriczko E. 2020 | Poland | children Age 8–10 (n=4,972 ) | child | cross-sectional | determine sociodemographic risk factors that predict an increase in BMI | parents’ body weight, physical activity, general health, birth weight, time spent watching TV, eating sweets, lower height in the mother |
| Views of City, County, and State Policy Makers About Childhood Obesity | Robbins R, et al. 2013 | USA | stakeholders n= 48 | child | qualitative | understanding of policy maker views on childhood obesity | dual-working parents, physical activity, limited access to healthful food and easy access to unhealthful food, direct-to-child marketing, poor nutrition in school meals, lack of opportunities for physical activity, culture of playing inside vs outside, video games, tv, internet, children’s preference |
| Identifying the views of adolescents on the drivers of obesity | Savona N, et al. 2021 | Netherlands, Norway, Poland, Portugal, UK | aged 16-18 (n=257) | adolescent | qualitative | identify young people's perceptions of the drivers of adolescent obesity | commercial drivers of unhealthy diets; mental health and unhealthy diets; social media use, body image and motivation to exercise |
| The Associations of Parenting Factors with Adolescent Body Mass Index | Schneider EM, et al. 2013 | USA | 70 parent-adolescent dyads | adolescent | cross-sectional | investigate parent factors associated with adolescent overweight | Parental risk perceptions, limit setting, and nurturance |
| Two-decade trends and factors associated with overweight and obesity | Shakya S, et al. 2023 | Nepal | adults age 18 -29 (n= 21,281) | adult | cross-sectional | Estimating long-term trends as well as examining demographic factors associated with overweight/ obesity | age, gender, married status, urban residence |
| Childhood obesity: Influencing factors and prevention strategies | Shamah-Levy T, et al. 2022 | Mexico | children 5-11 (n=45,216) | child | cross-sectional | describe factors which influence overweight and obesity | residing in urban localities, age and gender, consumption of fruits and vegetables |
| Overweight/Obesity: An Emerging Epidemic in India | Shannawaz M, Arokiasamy P. 2018 | India | women age 15-49 (n=90,000) | adult | cross-sectional | determine the levels, trends, differentials and determinants of overweight/obesity | unemployment, religion, gender, living in urban areas, education, SES, age |
| Prolonged financial stress predicts subsequent obesity | Siahpush M, et al. 2014 | AUS | adults (n=7,787) | adult | cross-sectional | assess the association of prolonged financial stress (FS) with subsequent obesity. | prolonged financial stress (FS) independent of income , lower education |
| Violent Crime, Physical Inactivity, and Obesity | Singleton CR, et al. 2023 | USA | adults age≥18 (n=798) | adult | cross-sectional | Investigating the association between violent crime rate and adult physical inactivity and obesity | Violent crime rate |
| An Examination of the Impact of Social and Cultural Traditions Contributing to Overweight and Obesity | Spinner JR. 2022 | USA | women age 35-50 (n=15) | adult | qualitative | exploring how social and cultural factors and social networks impact the weight | income, family network, peer network, region |
| Prevalence and Correlates of Overweight and Obesity | Stival C, et al. 2022 | Bulgaria, England, France, Germany, Greece, Ireland, Italy, Latvia, Poland, Portugal, Romania, and Spain | age 15 ≥ (in England ≥16 and in Ireland ≥18 years) ( n=10810) | adolescent and adult | cross-sectional | provide updated estimates on the prevalence and determinants of overweight and obesity | age, lower level of education, socioeconomic status |
| Nationally representative surveys show gradual shifting of overweight /obesity | Sutradhar I, et al. 2021 | Nepal | women age 15-49 (n= 21,717) | adult | cross-sectional | identify the trend of prevalence of overweight and obesity, along with their determinants | age, educational status, wealth index, place of residence, ecological zone, developmental region, number of household members, marital status and ethnicity |
| Trend Impact Analysis (TIA) of community-based futures study for pediatric obesity | Taghizadeh S, et al. 2023 | Iran | Children age 7-18  (n=939,956) | child and adolescent | quantitative and qualitative | conduct a community-based futures study for pediatric obesity | covid 19, widespread expansion of online educational programs, obesogenic environments, integrating education interventions, taxation of unhealthy and fast foods, appropriate sports environments |
| Obesity and its association with sociodemographic factors | Thurber KA, et al. 2018 | AUS | adults age≥45 (n=214,816) | adult | Cross-sectional | provide insight into factors relating to obesity | physical activity, screen time, education, remoteness, area-level disadvantage |
| Childhood obesity in Mexico: A critical analysis of the environmental factors | Turnbull B, et al. 2019 | Mexico | children age 8-12 (n=60), 24 of their mothers and 28 teachers | child | qualitative | understand childhood obesity and the complex factors at play | low physical activity, watching television, spending time on the computer, not access safe public spaces, insufficient physical education in public schools, food as a way to express love in the family, easily access cheap, processed food, going out daily for junk food as a family activity and a way to bond |
| The Associations between Body Mass Index , Dietary Behaviour and Nutrition-Related Parenting Practices | Vaitkeviciute J, Petrauskiene A. 2019 | Lithuania | children age 7-8 (n=3969) | child | cross-sectional | identify the associations between the BMI, dietary behavior and nutrition-related parenting practices | Consumption of unhealthy and more protein-rich food, increased control of unhealthy food consumption, encouragement and pressure to eat by parents, the use of food as a reward or punishment, and the lower importance of family mealtime |
| Social Determinants of Obesity and Stunting | Vale D, et al .2022 | Brazil | adolescents (n=16,556) | adolescent | cross-sectional | identify the prevalence of obesity and and its associations with social determinants of health | indifference or dissatisfaction with body image, eating breakfast, household size, studying in private schools, region, gender, age, highest nutritional risk eating pattern, dining at fast-food restaurants, eating while watching television or studying |
| Associations between family-related factors, breakfast consumption and BMI | Van Lippevelde W, et al. 2013 | Eight European countries | children age 10-12  (n = 6374) | child | cross-sectional | investigate associations of family-related factors with children's breakfast consumption and BMI | Eating breakfast together, allowing to skip breakfast and negotiating about breakfast products |
| Factors Contributing to the Change in Overweight/Obesity Prevalence | Verma M, et al. 2023 | India | Adults age 15–49 (n=1,291,449) | adult | cross-sectional | estimating the contribution of different socio-demographic factors to the increasing overweight/obesity | age, improving socio-economic status, smoking, unclean cooking fuel, diabetes |
| Association between the school environment and children's BMI | Wafa SW, Ghazalli R. 2020 | Malaysia | 32 teachers, school children age 9- 11 (n=400) | child | cross-sectional | investigate the association between school environmental factors and obesity | health professional involvement, simple exercise before class, encouragement to walk/ride bicycle to/from school, no high-calorie food sold, healthy options of foods and drinks at tuck shop, availability of policy on physical activity and training teacher as a role model. |
| Dimensions of national culture associated with different trajectories of mean BMI | Wallace C, et al .2019 | International (87 countries) | NA | NA | cross-sectional | quantified longitudinal associations between six dimensions of national culture mean population BMI | individualistic, uncertainties or ambiguities, focus on freedom and happiness (higher Indulgence), focus on short-term goals, intolerant of inequalities and power hierarchies, less competitive and more feminine (lower Masculinity). |
| Trends in obesity by socioeconomic status | Wariri O, et al. 2021 | 11 countries in sub-Saharan Africa | women age 15-49 | adult | cross-sectional | tracked and described obesity trends across multiple equity dimensions | wealth, education, urban dwellers, availability of non-traditional foods, attraction for western products, cultural beliefs in Africa that a bigger body is admirable and a sign of wealth, subside, tax, strong public health regulations on the numbers, availability and distribution of high-calorie fast food restaurants |
| Prevalence and determinants of overweight and obesity | Woronko C, et al. 2023 | Canada | child-mother dyads (n=275) | child | longitudinal | Estimating the prevalence of overweight/obesity and identifying the determinants of BMI | primiparity, high birth weight, accelerated weight gain during infancy and early introduction of solid foods |
| Significantly different roles of economic affluence in sex-specific obesity prevalence rates | You W, Henneberg M. 2022 | International (191 countries) | age ≥18 | adult | cross-sectional | examining the difference between these two relationships (socio-economic status and prevalence of obesity) | gross domestic product (GDP) per capita |
| Determinants of obesity in population of PURE study from Lower Silesia | Zdrojowy-Wełna A, et al. 2018 | Poland | adults age 30–80 (n=1,064) | adult | Cross-sectional | investigate environmental and individual determinants of obesity | rural inhabitancy, chronic medication, unemployment, age, sedentary leisure time activity, hypertension and diabetes in family, family related stress |
| Environment- and epigenome-wide association study of obesity | Zhao J, et al. 2023 | Hong Kong | Child age ~11.5 (n = 8327) | child | longitudinal | providing more insight about the role of environmental exposures and epigenetics in early life obesity | Maternal second-hand smoking, maternal weight, and birth weight, dairy intake, artificially sweetened beverages, physical activity, snoring, binge eating, and earlier puberty |
| Prevalence of Overweight and Obesity, and Its Associated Factors | Zubery D, et al. 2021 | Tanzania | adults age 18–60 (n=305) | adult | cross-sectional | identify the prevalence of overweight and obesity and its associated risk factors | Age, gender, marital status, years spent with the current institution, using private car or public transport to and from work, sedentary work |
